# Breastfeeding vs. breast milk transmission during COVID-19 pandemic, which is more important?

**DOI:** 10.3389/fped.2023.1253333

**Published:** 2023-09-06

**Authors:** Yan-fei He, Jun-qiang Liu, Xiao-dong Hu, Hu-ming Li, Ni Wu, Jie Wang, Zhi-gang Jiang

**Affiliations:** ^1^ Health Management Center, The Sixth Medical Center, Chinese PLA General Hospital, Beijing, China; ^2^ Department of Thoracic Surgery, The Sixth Medical Center, Chinese PLA General Hospital, Beijing, China; ^3^ Department of Endocrinology, The Sixth Medical Center, Chinese PLA General Hospital, Beijing, China; ^4^ Department of Respiratory Medicine, The Sixth Medical Center, Chinese PLA General Hospital, Beijing, China; ^5^ Department of Statistics, Zunyi Medical University, Zunyi, China

**Keywords:** breast milk, breastfeeding, SARS-CoV-2, COVID-19, lactation, vertical transmission, antibodies

## Abstract

The catastrophic coronavirus disease 2019 (COVID-19) pandemic has raised many health questions, and whether breast milk from SARS-CoV-2 infected mothers may be a vector for SARS-CoV-2 transmission has become a hot topic of concern worldwide. Currently, there are extremely limited and conflicting data on the risk of infection in infants through breastfeeding. For this reason, we investigated almost all current clinical studies and systematically analyzed the presence of SARS-CoV-2 and antibodies in the breast milk of mothers infected with SARS-CoV-2, their effects on newborns, and the mechanisms involved. A total of 82 studies were included in this review, of which 66 examined the presence of SARS-CoV-2 in breast milk samples from mothers diagnosed with COVID-19, 29 reported results of antibody detection of SARS-CoV-2 in breast milk, and 13 reported both nucleic acid and antibody test results. Seventeen studies indicated the presence of detectable SARS-CoV-2 nucleic acid in breast milk samples, and only two studies monitored viral activity, both of which reported that infectious viruses could not be cultured from RNA-positive breast milk samples. All 29 studies indicated the presence of at least one of the three antibodies, IgA, IgG and IgM, in breast milk. Five studies indicated the presence of at least one antibody in the serum of breastfed newborns. No COVID-19-related deaths were reported in all 1,346 newborns. Our study suggests that direct breastfeeding does not pose an additional risk of infection to newborns and that breast milk is a beneficial source of anti-SARS-CoV-2 antibodies that provide passive immune protection to infants. In addition, direct breastfeeding would provide maternal benefits. Our review supports the recommendation to encourage direct breastfeeding under appropriate infection control guidelines.

**Systematic Review Registration:** https://www.crd.york.ac.uk/PROSPERO/#myprospero, identifier: 458043.

## 1. Introduction

During the global pandemic of COVID-19, the safety of direct breastfeeding by infected mothers to their infants has become one of the hottest topics. Few conditions are considered to be clear contraindications to breastfeeding. Known pathogens that can be transmitted in breastfeeding include HIV (1), human T-lymphotropic virus 1 (2), and cytomegalovirus (3). Previously, some studies showed the presence of SARS-CoV-2 in the breast milk of a few cases (4–6), a situation that caused considerable alarm. There is insufficient evidence as to whether breastfeeding is a possible mode of vertical mother-to-child transmission.

Breast milk is the gold standard for infant feeding. Not only does breastfeeding provide the best source of nutrition for the newborn and a powerful first barrier against infection, but it also has the emotional stimulation to enhance dynamic, two-way communication between mother and infant, laying the foundation for a physical and psychological bond between mother and child (7). In the short and long term, breastfeeding provides tremendous health benefits for both child and mother, especially the immunological properties of breast milk that make it a protective factor against infant morbidity and mortality (8). For infants, passive immunity is derived mainly from breast milk. SARS-CoV-2-specific antibodies have been reported to be detected in the breast milk of infected women (9, 10). The transfer of these antibodies to infants through breast milk may protect against SARS-CoV-2 infection. So, are SARS-CoV-2 antibodies present in breast milk or not? Could they provide benefits to newborns? Although the World Health Organization declared on May 5, 2023, that the spread of COVID-19 was no longer a “public health emergency of international concern”, the global interest in ensuring that mothers and infants are not separated and that breastfeeding mothers are provided with the necessary support and/or breast milk for their infants has been well established for some time, and there are very limited and conflicting data on the presence of SARS-CoV-2 in breast milk and the potential for vertical transmission in breast milk. Therefore, there is a strong need for a comprehensive analysis and discussion of the evidence regarding the potential for vertical transmission of breastfeeding and the presence of anti-SARS-CoV-2 antibodies in breast milk. Here, we plan to use this systematic review to synthesize the data published to date on the risk of SARS-CoV-2 transmission through breast milk, to provide a comprehensive summary of the presence and characteristics of antibodies in breast milk and their effects on infants, to provide evidence for assessing the risk-benefit of breast milk transmission vs. breastfeeding, and to provide a basis for the management of mother-infant dyads and optimal health strategies for breastfeeding in similar outbreaks in the future.

## 2. Methodology

### 2.1. Identify research question

Is breast milk of SARS-CoV-2 infected mothers a possible carrier of SARS-CoV-2 transmission? What is more important, breastfeeding or breast milk transmission?

### 2.2. Identify relevant types of evidence

An experienced information specialist conducted a comprehensive search of PubMed, MEDLINE, CNKI, Wanfang Database, bioRxiv, medRxiv, Embase, and Cochrane Library online databases, and to maximize the scope of the review without time and language restrictions, the last data update was April 30, 2023. We used the keywords “SARS-CoV-2”, “COVID-19”, “breast milk”, and “antibody” individually or in combination to achieve a comprehensive literature search. We also searched the gray literature of the preprint servers bioRxiv and medRxiv. In addition, we manually searched the references of the original articles included in the study to avoid missing important literature that was not noted in the initial search. Inclusion criteria were (i) the presence of SARS-CoV-2 RNA or/and the presence of anti-SARS-CoV-2 antibodies in mothers diagnosed with SARS-CoV-2 infection and (ii) breast milk samples. Studies that included the same study population but reported different data and outcomes were also included. Exclusion criteria were (i) questionnaires; (ii) studies that explored breastfeeding intentions only (psychological); (iii) studies in which mothers were not diagnosed with SARS-CoV-2 infection; (iv) artificial vaccinations or mixed groups with vaccinations that could not be grouped separately; (v) studies with severely missing data on mothers, breast milk samples, or newborns; and (vi) studies that tested only breast milk studies that detect factors other than SARS-CoV-2 RNA or antibodies in breast milk, including cytokines, chemical elements, proteomes, and nerve growth factors. None of the literature types were restricted. See Supplementary Material S1 for detailed search strategies.

### 2.3. Study selection

After completing the initial search, two independent reviewers conducted a screening process, and literature with quantifiable evidence was included in our review. We excluded duplicate publications and duplicate case studies (we identified duplicates based on author name, location, participant admission date, maternal and neonatal characteristics, and publication date). One reviewer reviewed the selected articles in their entirety, and studies that contained complete data descriptions were used for data graphing. Any conflicts that arose during the data extraction process were resolved by consensus with a third reviewer. All seven authors participated in the discussion and decided on the topic.

### 2.4. Data extraction

**Figure 1:**
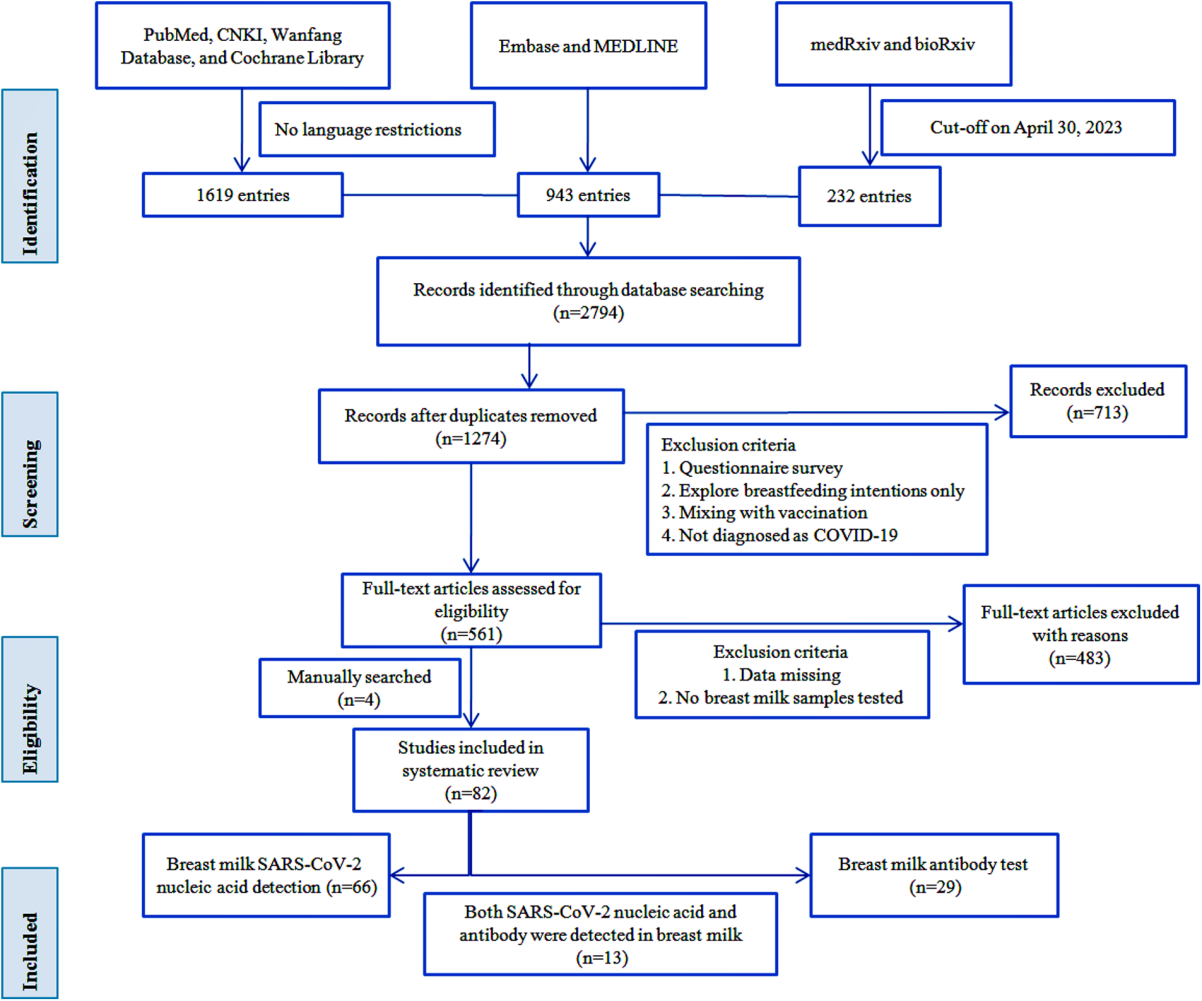
Flow diagram of literature search.

As of April 30, 2023, we retrieved a total of 1,274 publications, and after screening by the inclusion criteria described above, we qualified 561 full-text papers, plus four manually retrieved papers, resulting in 82 papers for inclusion in this review. We extracted data for each paper regarding the first author's name, country, study design, basic demographic characteristics of participants, maternal samples, breast milk samples, SARS-CoV-2 RNA or antibody test results, and number of newborns and feeding patterns for tabulation and discussion. We did not perform any meta-analysis of the data obtained but used a narrative synthesis because, as expected, there was substantial heterogeneity among the studies we retrieved, making it difficult to make meaningful comparisons between studies. Figure 1 shows a visual representation containing the workflow.

## 3. Results

A total of 82 studies were finally included (4, 6, 9–88), including three studies published in Chinese (25, 27, 28). They came from 21 countries, with 26 studies from China (31.7%). The earliest study start time is December 2019 (24), and the longest study span is 12 months (86). Thirteen studies reported both nucleic acid test results and antibody test results. Of 82 studies, 33 case reports and nine case series were included. A total of 1,755 mothers and 1,346 newborns and at least 2,043 breast milk samples were included in the studies. The maximum sample size of mothers included in the study was 165, the maximum sample size of breast milk was 316, and the maximum sample size of newborns was 165. The follow-up period ranged from three days to 11 months. The characteristics of the included studies are listed in Table 1.

**Table 1:** Characteristics of the included studies.

Study	Study time span	Country	Type of study	Maternal number	Breast milk samples (*n*)	Neonatal number	Infant sex (M/F, *n*)	Delivery mode cesarean vaginal		Follow-up time
Groß et al. (4)	Article published online May 21, 2020	Germany	Case report	2	11	2	NA	NA	NA	26 days
Chambers et al. (6)	Between March 27 and May 6, 2020	United States	Case series	18	64	NA	NA	NA	NA	2 months
Peng et al. (9)	Between February and April, 2020	China	Longitudinal study	24^a^	44	25	15/10	21	3	80 days
Demers-Mathieu et al. (10)	Between April and November, 2020	United States	Prospective cohort study	7^a^	7	7	4/3	NA	NA	7 months
Hinojosa-Velasco et al. (11)	May, 2021	Mexico	Case report	1	2	1	0/1	1	0	13 days
Olivini et al. (12)	March, 2020	Italy	Retrospective observational case series	5	2	5	3/2	3	2	1 month
Sahin et al. (13)	Between March 11 and June 11, 2020.	Turkey	Prospective cohort study	29	10	10	NA	5	5	3 months
Lugli et al. (14)	Article published online August 25, 2020	Italy	Case report	1	2	1	0/1	1	0	32 days
Cui et al. (15)	February, 2020	China	Case report	1	3	1	0/1	NA	NA	1 month
Gómez-Torres et al. (16)	Between April and July, 2020	Spain	Prospective study	37	197	37	NA	13	26	5 weeks
Zhu et al. (17)	Between February 1 and March 25, 2020	China	Case report	5	8	5	NA	4	1	41 days
Kunjumon et al. (18)	Between May and October, 2020	United States	Prospective observational study	19	19	19	13/6	6	13	1 month, *n* = 18; 6 months, *n* = 1
Kam et al. (19)	February 2020	Singapore	Case report	1	1	1	1/0	NA	NA	18 days
Lang et al. (20)	February 2020	China	Case report	1	≥2	1	1/0	1	0	14 days
Yan et al. (21)	March, 2020	China	Expanded case series	116	12	100^b^	NA	85	14	2 months
Chen et al. (22)	January, 2022	China	Retrospective observational case series	9	6	9	NA	9	0	14 days
Liu et al. (23)	Between January 31 and February 29, 2020	China	Prospective observational study	19	10	19	NA	18	1	1 month
Chen et al. (24)	Between December 8, 2019 and March 20, 2020	China	Prospective observational study	118	3	70	NA	63^c^	5	74 days
Zhuang et al. (25)	January, 2022	China	Case report	1	1	1	1/0	1	0	14 days
Mao et al. (26)	February, 2020	China	Case report	1	1	1	1/0	NA	NA	1 month
Lei et al. (27)	February, 2020	China	Case series	9	4	4	NA	3	1	37 days
Chen et al. (28)	Between January 19 and February 10, 2020	China	Case series	3	1	1	NA	1	0	23 days
Wu et al. (29)	Between 31 January and 9 March 2020.	China	Retrospective cohort study	13	3	5	NA	4	1	37 days
Piersigilli et al. (30)	March, 2020	Belgium	Case report	1	1	1	0/1	1	0	28 days
Wang et al. (31)	February, 2020	China	Case report	1	1	1	1/0	1	0	17 days
Tam et al. (32)	March, 2020	Australia	Case report	1	7	1	1/0	NA	NA	66 days
Bertino et al. (33)	Between April 1 and July 31, 2020	Italy	prospective collaborative observational study	14	14	14	NA	9	5	1 month
Dong et al. (34)	Between January 28 and February 28, 2020	China	Case series	7^a^	7^a^	7^a^	NA	2	5	1 month
Prasad et al. (35)	Article received 16 March 2021	India	Prospective observational study	50	49	50	NA	50	0	1 month
Costa et al. (36)	March, 2020	Italy	Case report	2	6^i^	NA	NA	2	0	11 days
Yin et al. (37)	Between January 28 and February 28, 2020	China	Retrospective cohort study	31	14	17	NA	13	4	36 days
Kilic et al. (38)	Between 11 March 2020 and 31 January 2021	Turkey	Prospective observational study	15	26	15	NA	5	10	20 days
Thanigainathan et al. (39)	Between April and August, 2020	India	Descriptive study	30	31^d^	31	NA	NA	NA	10 days
Li et al. (40)	February, 2020	China	Case report	1	1	1	1/0	1	0	18 days
Perrone et al. (41)	Between March and April, 2020	Italy	Case report	1	1	1	0/1	0	1	39 days
Kalafat et al. (42)	March, 2020	Turkey	Case report	1	1	1	1/0	1	0	10 days
Salvatori et al. (43)	April, 2020	Italy	Case series	2	2	2	1/1	NA	NA	5 days
Alzaghal et al. (44)	March, 2020	Jordan	Case report	1	1	1	0/1	1	0	18 days
Marín et al. (45)	Article published 2020	Spain	Observational prospective study	7	7	7	1/6	1	6	NA
Buonsenso et al. (46)	March, 2020	Italy	Observational study	7	20	2^e^	1/1	2	0	18 days
Krogstad et al. (47)	Between March and September, 2020	United States	Observational cohort study	66^a^	118	64	29/35	NA	NA	97 days
Hall et al. (48)	Between May and April, 2021	Austria	Retrospective study	118	44	100	NA	44	56	NA
Takahashi et al. (49)	Article accepted March 11, 2021	Japan	Case report	1	3	1	1/0	1	0	1 month
Fan et al. (50)	Between January and February, 2020	China	Case series	2	2	2	*F*, *n* = 1	2	0	1 month
Liu et al. (51)	February, 2020	China	Case report	1	1	1	1/0	0	1	1 month
Chu et al. (52)	Between January and February, 2020	China	Case report	1	2	1	1/0	1	0	1 month
Dong et al. (53)	Between January and February, 2020	China	Case report	1	1	1	0/1	1	0	51 days
Mattar et al. (54)	Between March and August, 2020	Singapore	Prospective observational study	16	2	5	NA	0	5	80 days
Kirtsman et al. (55)	Article published online May 14, 2020	Canada	Case report	1	1	1	1/0	1	0	30 days
Han et al. (56)	March, 2020	South Korea	Case report	1	1	1	0/1	0	1	18 days
Peng et al. (57)	March, 2020	Canada	Case report	1	8	1	0/1	1	0	14 days
Xiong et al. (58)	Between March, 2020	China	Case report	1	1	1	1/0	0	1	40 days
Bastug et al. (59)	April, 2020	Turkey	Case report	1	3	1	1/0	0	1	14 days
Schoenmakers et al. (60)	April, 2020	Netherlands	Case report	1	2	1	0/1	1	0	3 days
Sharma et al. (61)	Between April 1 and August 31, 2020	India	Ambispective observational study	41	23	44	24/20	23	18	5 months
Luo et al. (62)	Between February 1 and March 15, 2020	China	Observational study	14^a^	14	14	NA	12	2	54 days
Dong et al. (63)	Between February 26 and April 9, 2020	China	Case report	1	7^h^	1	0/1	1	0	42 days
De Socio et al. (64)	March, 2020	Italy	Case report	1	1	1	NA	0	1	15 days
Young et al. (65)	Between December 2020 and May 2021	United States	Prospective study	47^a^	47	47	24/23	NA	NA	90 days
Calabretto et al. (66)	Between May and December, 2021	Italy	Ambispective observational study	12	12	NA	NA	NA	NA	7 months
Pace et al. (67)	Between April and December, 2020	United States	Multicenter longitudinal study	64	316	53	NA	NA	NA	8 months
Bäuerl et al. (68)	Between April and December, 2020	Spain	Prospective, multicentre longitudinal study	60^a^	60	55^f^	24/30^g^	13	42	8 months
Walczak et al. (69)	March, 2020	Australia	Case report	1	1	1	NA	0	1	10 days
Yu et al. (70)	February, 2020	China	Case report	1	4	1	1/0	NA	NA	28 days
Gao et al. (71)	Between January 19 and April 5, 2020	China	Ambispective observational clinical analysis	14	12	14	NA	12	2	46 days
Fenizia et al. (72)	Between March and April, 2020	Italy	Prospective multicenter study	31	11	31	18/13	6	25	NA
Pace et al. (73)	Between March 2020 and September 2020	United States	Prospective study	18	37	18	9/9	6	12	15 days
Juncker et al. (74)	Article published December 14, 2021	Netherlands	Longitudinal cohort study	18^a^	82	18	11/7	6	12	70 days
Conti et al. (75)	Between November 2020 and May 2021	Italy	Observational cohort study	28	48 h, *n* = 6; 2 months, *n* = 10	30	18/12	14	14	2 months
van Keulen et al. (76)	May, 2020	Netherlands	Prospective cohort study	29^a^	29	29	NA	NA	NA	13 weeks
Lebrão et al. (77)	Article received June 22, 2020	Brazil	Case report	1	2	1	0/1	1	0	45 days
Favara et al. (78)	Between April and October, 2020	United Kingdom	Case report	1	2	1	NA	NA	NA	6.5 months
Fox et al. (79)	April, 2020	United States	Cross-sectional observational study	8^a^	8	NA	NA	NA	NA	None
Juncker et al. (80)	Between October 12, 2020 and February 24, 2021	Netherlands	Prospective cohort study	165^a^	165	NA	NA	NA	NA	10 months
Szczygioł et al. (81)	Between February 15 and May 1, 2021	Poland	Observational study	72^a^	72	72	39/33	47	25	8 months
Bode et al. (82)	Between March 14 and September 1, 2020	United States	Observational study	21	21	22	11/11	NA	NA	8 months
Bobik et al. (83)	Between July and October, 2020	Russia	Observational study	41	41	35	NA	NA	NA	3 months
Duncombe et al. (84)	Article published online July 18, 2021	United States	Prospective cohort study	2^a^	2	NA	NA	NA	NA	6 months
Juncker et al. (85)	Between May and August, 2020	Netherlands	Longitudinal Follow-Up Study	29^a^	66	29	NA	NA	NA	5 months
Dutra et al. (86)	Between June 2020 and May 2021	Brazil	Cross-sectional study	165	165	165	76/89	80	85	11 months
Narayanaswamy et al. (87)	Between March and September, 2020	United States	Observational study	15	15	NA	NA	NA	NA	7 months
Wachman et al. (88)	Between July 2020 and May 2021	United States	Prospective study	31	31	6	14/17	14	17	6 weeks
TOTAL				1755	≥2,043	1,346				

M/F, male/female; NA, not available.

^a^ RT-PCR-confirmed COVID-19 cases.

^b^ Including 1 pair of twins.

^c^ Including 2 sets of twins.

^d^ The sample of the positive breast milk was retested the next day.

^e^ Two of the seven pregnant women delivered.

^f^ Missing data from 5 individuals.

^g^ Missing data from 6 individuals.

^h^ One breast milk was tested for nucleic acid and six breast milk was tested for antibodies.

^i^ The breast milk samples were all from the same mother.

### 3.1. Results of SARS-CoV-2 nucleic acid detection in breast milk

A total of 66 studies examined the presence of SARS-CoV-2 in breast milk samples from mothers with a confirmed diagnosis of COVID-19 (4, 6, 9, 11–73). All included studies used RT-PCR assays. Fifty-five studies reported the timing of breast milk specimen collection, with the earliest being immediately after delivery and the longest span of breast milk specimen collection being 206 days. A summary of studies on the detection of SARS-CoV-2 in human milk is presented in Table 2.

**Table 2:** Characteristics of included studies of detection SARS-CoV-2 in human breast milk.

	Maternal characteristics							Infant characteristics								
Study	Number of pregnant women	Maternal age [Mean/±SD, (range), Years]	Time of milk collection	Breast milk RT-PCR test results (*n*)		Feeding method	Detection method	Number of newborns	Birth weight [Mean/±SD, (range), grams]	Gestational age at delivery [Mean/±SD, (range), weeks]	Apgar score		Nasopharyngeal swab RT-PCR test results (*n*)		Neonatal death	Findings and conclusions
				Positive	Negative						1 min	5 min	Positive	Negative		
Groß et al. (4)	2	NA	Day 10, 12, 13, 14, 23 and 25 after delivery	4^q^	7	Breastfeeding	RT-PCR	2	NA	NA	NA	NA	2	0	Discharge	SARS-CoV-2RNA is detectable in breast milk and the possibility of transmission of the virus through breastfeeding requires further investigation.
Chambers et al. (6)	18	34.4 ± 5.2	0–41 days after the onset of symptoms	1	63	NA	RT-PCR	NA	NA	From birth to 19 months (postpartum)	NA	NA	NA	NA	NA	SARS-CoV-2 RNA does not represent replication-competent virus and that breast milk may not be a source of infection for the infant.
Peng et al. (9)	24	29.8 ± 3.7	The day of delivery, as well as the 3rd, 7th, 14th, 21st, 28th, 35th, 42nd, 56th and 70th day post-delivery	0	44	Breastfeeding, *n* = 14; formula feeding, *n* = 10	RT-PCR	25	3.0 ± 0.5	38.2 ± 2.1	9.2 ± 0.8	9.8 ± 0.4	NA	NA	No death reported	All 44 breast milk samples were negative for SARS-CoV-2 nucleic acid.
Hinojosa-Velasco et al. (11)	1	21	4 days after delivery	1	1	Formula	RT-PCR	1	3,075	38	8	9	1	0	Discharge	Neonates are at risk of being infected with SARS-CoV-2 during breastfeeding.
Olivini et al. (12)	5	27–36	13 and 23 days after delivery	0	2	Breastfeeding	RT-PCR	5	2,750–4,440	36–41	9, *n* = 4; 5, *n* = 1	10	4	1	Discharge, *n* = 4; transferred, *n* = 1	Neonatal COVID-19 appears to have a horizontal transmission.
Sahin et al. (13)	29	17–40	NA	0	10	NA	RT-PCR	10	1,630–4,010	31–40	8	9	0	10	No death reported	Samples of breast milk were negative for SARS-CoV-2.
Lugli et al. (14)	1	33	6 days	2	0	Breastfeeding	RT-PCR	1	1,614	32	3	6	0	1	Discharge	A healthy premature infant was inadvertently breastfed SARS-CoV-2-positive breast milk by his mother, but was not infected.
Cui et al. (15)	1	NA	55–57 days after delivery	0	3	Breastfeeding	RT-PCR	1	NA	55 days (postpartum)	NA	NA	1	0	Discharge	Samples of breast milk were negative for SARS-CoV-2.
Gómez-Torres et al. (16)	37	33.9 ± 5.4	1st week and 5th week postpartum	0	197	NA	RT-PCR	37	3,187 ± 543	39.1 ± 1.7	NA	NA	0	37	Well	No SARS-CoV-2 mRNA was detected in human milk samples.
Zhu et al. (17)	5	32 (27–34)	2 and 3 days after delivery	2^f^	6	NA	RT-PCR	5	NA	35–40	NA	NA	NA	NA	Well	Detectable SARS-CoV-2 nucleic acid in human breast milk from a puerperal woman with COVID-19.
Kunjumon et al. (18)	19	26.94 ± 5.07	1–3 days after admission	1	18	Breastfeeding	RT-PCR	19	3,293 ± 503	38.70 ± 1.73	9	9	0	19	Well	The majority of breast milk samples (95%) did not contain detectable viruses and there was no evidence of SARS-CoV-2 infection in breastfed newborns.
Kam et al. (19)	1	NA	11 days after maternal symptoms onset	0	1	NA	RT-PCR	1	NA	6 months (postpartum)	NA	NA	1	0	Well	There is no evidence of vertical transmission through breast milk.
Lang et al. (20)	1	30	Beginning on the fourth day of hospitalization	0	≥2	Breastfeeding	RT-PCR	1	NA	35.7	9	10	0	1	Discharge	Repeated RT-PCR analyses of the mother's breast milk were consistently negative for SARS-CoV-2.
Yan et al. (21)	116	30.8 (24–41)	At first lactation, not detailed	0	12	NA	RT-PCR	100	3,108 ± 526	38.4 [37.3, 39.4]^a^	9	10	14	86	Well^d^	Breastfeeding does not appear to increase the risk of mother-to-child transmission of SARS-CoV-2.
Chen et al. (22)	9	26–40	At first lactation, not detailed	0	6	NA	RT-PCR	9	1,880–3,820	36–39	8–9	9–10	0	9	No death reported	There is no evidence of vertical transmission of breast milk in women with COVID-19.
Liu et al. (23)	19	26–38	At first lactation, not detailed	0	10	Formula	RT-PCR	19	3,293 ± 425	38.6 ± 1.5	8	9	0	19	Well	Vertical transmission of SARS-CoV-2 through breast milk has not been found.
Chen et al. (24)	118	31	NA	0	3	NA	RT-PCR	70	NA	NA	8–9	NA	0	8^c^	Well	Samples of breast milk were negative for SARS-CoV-2.
Zhuang et al. (25)	1	33	5 days after delivery	0	1	Formula	RT-PCR	1	2,870	37.3	8	10	0	1	Discharge	Vertical transmission of SARS-CoV-2 through breast milk has not been found.
Mao et al. (26)	1	NA	NA	0	1	Breastfeeding	RT-PCR	1	NA	14 months (postpartum)	NA	NA	1	0	Discharge	Vertical transmission of SARS-CoV-2 through breast milk has not been found.
Lei et al. (27)	9	29 (24–35)	NA	0	4	NA	RT-PCR	4	2,350–3,400	34–37^b^	9	10	0	4	Well	The breast milk samples collected from the infected mother was negative for SARS-CoV-2 by RT-PCR.
Chen et al. (28)	3	30–38	1–5 days after delivery	0	1	Breastfeeding	RT-PCR	1	2,670	35^b^	9	10	0	1	Well	The risk of transmission of COVID-19 through breast milk is low.
Wu et al. (29)	13	27–29	Day 1, 6 and 27 after delivery	1	2	NA	RT-PCR	5	2,300–3,910	35–38	7–9	9–10	0	5	No death reported	Vertical transmission of SARS-CoV-2 to newborns through breast milk is unlikely.
Piersigilli et al. (30)	1	NA	Before the seventh day after delivery	0	1	Breastfeeding	RT-PCR	1	960	26.6	5	8	1	14-day postpartum	Well	There is likely to be horizontal transmission from mother to baby.
Wang et al. (31)	1	34	1 day after delivery	0	1	Formula	RT-PCR	1	3,205	40	8	9	1	0	Discharge	Breast milk nucleic acid negative for SARS-CoV-2, while newborn nucleic acid positive for SARS-CoV-2.
Tam et al. (32)	1	40	Day 5, 9, 10, 11, 12, 13 and 15 after symptoms onset	2	5	Breastfeeding	RT-PCR	1	NA	32	NA	NA	0	1	Discharge	Finding SARS-CoV-2 RNA in the breast milk sample does not indicate a viable virus.
Bertino et al. (33)	14	24–38	1 and 2 days after delivery	1	13	Breastfeeding	RT-PCR	14	NA	30–41	NA	NA	4	10	Well	SARS-CoV-2 positive mothers do not expose their newborns to an additional risk of infection by breastfeeding.
Dong et al. (34)	7	NA	Collected immediately after delivery	0	7	NA	RT-PCR	7	NA	38–42	8	9	0	7	No death reported.	There is no evidence that SARS-CoV-2 is transmitted vertically to infants through breast milk.
Prasad et al. (35)	50	NA	within 4 days of delivery	0	49	Breastfeeding	RT-PCR	50^d^	NA	NA	NA	NA	2	48	No death reported.^d^	All the samples of breast milk were negative for SARS-CoV-2.
Costa et al. (36)	2	38–42	1 day after delivery	3^e^	3^e^	Formula	RT-PCR	2	NA	35.7	NA	NA	0	2	No death reported.	Intermittent viral excretion may be present in breast milk.
Yin et al. (37)	31	31.0 ± 4.3	NA	0	14	NA	RT-PCR	17	2,450–4,100	35–41	8	9	0	17	No death reported.	There was no evidence that SARS-CoV-2 could be transmitted from COVID-19 mothers to infants during pregnancy.
Kilic et al. (38)	15	24–44	within 2 days after maternal symptoms onset	4	22	Breastfeeding, *n* = 12	RT-PCR	15	NA	From 1 to 300 days (postpartum)	NA	NA	8	7	Well	SARS-CoV-2 can be excreted into human milk, however, this does not indicate that SARS-CoV-2 is infectious and that human milk may be a source of infection for infants.
Thanigainathan et al. (39)	30	NA	Between 48 and 72 h after delivery	1	30^f^	Breastfeeding	RT-PCR	30	NA	NA	NA	NA	0	30	Well	No evidence of SARS-CoV-2 transmission to children through breast milk was found.
Li et al. (40)	1	30	1 day after delivery	0	1	NA	RT-PCR	1	NA	35	NA	NA	0	1	Discharge	Vertical transmission of SARS-CoV-2 from mother to child via breast milk seems unlikely.
Perrone et al. (41)	1	NA	11 days after delivery	0	1	Breastfeeding	RT-PCR	1	NA	32	NA	NA	0	1	Discharge	Maternal breast milk from COVID-19 was negative for SARS-CoV-2 by RT-PCR.
Kalafat et al. (42)	1	32	NA	0	1	NA	RT-PCR	1	2,790	35.4	NA	9	0	1	No death reported.	Maternal breast milk from COVID-19 was negative for SARS-CoV-2 by RT-PCR.
Salvatori et al. (43)	2	31	NA	0	2	Breastfeeding	RT-PCR	2	3,200–5,200	39–42	NA	NA	2	0	No death reported.	Horizontal transmission of SARS-CoV-2 from mother to newborn may occur through respiratory droplets rather than through breast milk.
Alzaghal et al. (44)	1	30	NA	0	1	Breastfeeding	RT-PCR	1	2,500	36	8	9	0	3	Discharge	Breast milk tested negative for SARS-COV-2 in SARS-COV-2-infected patients.
Marín et al. (45)	7	31–37	26–48 h after delivery	0	7	Breastfeeding	RT-PCR	7	2,788–4,574	38–41	3–9	9–10	0	7	No death reported	Breast milk was not a source of SARS-CoV-2 transmission.
Buonsenso et al. (46)	7	38–42	Day 1, 3, 11, 14 and 17 after delivery	3^g^	17	Formula	RT-PCR	2	2,300–3,390	35–38	8–9	9–10	1^h^	2^i^	Well	Although SARS-CoV-2 was detected in breast milk, it is unlikely to be transmitted to the newborn.
Krogstad et al. (47)	66	35.8	1–189 days after maternal symptoms onset	7^o^	59^o^	Breastfeeding	RT-PCR	64	NA	7.97 months (postpartum)	NA	NA	NA	NA	No death reported	Even if the SARS-CoV-2 PCR test was positive, there was no evidence of infectious SARS-CoV-2 in the milk of recently infected women.
Hall et al. (48)	118	17–43	NA	0	44	NA	RT-PCR	100	1,045–4,360	29–39	≤7, *n* = 14	NA	2^j^	100^k^	No death reported.	There was no virus found in breast milk.
Takahashi et al. (49)	1	27	Day 1, 2, and 3 after birth	0	3	Breastfeeding	RT-PCR	1	3,140	38	8	9	0	1	Well	Vertical transmission of SARS-CoV-2 is presumed rare.
Fan et al. (50)	2		After delivery	0	2	Formula	RT-PCR	2	2,890–3,400	36–37	9	10	0	2	Discharge	There is a low risk of vertical transmission through breast milk.
Liu et al. (51)	1	33	1 day after maternal symptoms onset	0	1	Breastfeeding	RT-PCR	1	NA	39	NA	NA	0	1	Well	Breastfeeding may be less of a risk than anticipated.
Chu et al. (52)	1		31 and 32 days after delivery	0	2	Breastfeeding	RT-PCR	1	NA	NA	NA	NA	NA	NA	Well	A lactating woman with COVID-19 whose breast milk was negative for SARS-CoV-2 RNA breastfed her child and no transmission occurred.
Dong et al. (53)	1	29	6 days after delivery	0	1	Formula	RT-PCR	1	3,120	34.3	9	10	0	1	Discharge	Breast milk of a mother with COVID-19 was negative for SARS-CoV-2 RNA.
Mattar et al. (54)	16	23–36	Colostrum samples	0	2	Breastfeeding	RT-PCR	5	NA	38–41	NA	NA	0	5	Discharge	There is no clear evidence of mother-to-child transmission through breast milk in COVID-19 women who give birth.
Kirtsman et al. (55)	1	40	2 days after delivery	1	0	Breastfeeding	RT-PCR	1	2,930	35.7	9	9	1	0	Discharge	Nasopharyngeal swabs from mothers, breast milk and nasopharyngeal swabs from newborns were positive for SARS-CoV-2 RNA, but contamination of breast milk with respiratory secretions could not be excluded.
Han et al. (56)	1	NA	10 days after delivery	0	1	Breastfeeding	RT-PCR	1	3,730	38	NA	NA	1	0	Discharge	SARS-CoV-2 was not detected in breast milk. The infant got infected from family member.
Peng et al. (57)	1	25	At day 2, 3, 4, 5, 6, 7, 10 and 14 of delivery	0	8	NA	RT-PCR	1	2,600	35.4	9	10	0	1	Discharge	The risk of mother-to-child transmission of SARS-CoV-2 through breast milk is extremely low.
Xiong et al. (58)	1	25	On the day of delivery	0	1	NA	RT-PCR	1	3,070	38	10	10	0	1	Discharge	No SARS-CoV-2 RNA was found in the breast milk of a pregnant woman with COVID-19 and her neonate was not infected with SARS-CoV-2.
Bastug et al. (59)	1	20	At day 1, 3 and 4 after delivery	3	0	Breastfeeding	RT-PCR	1	2,980	39	NA	NA	1 (4 days after delivery)	0 (8–10 h after birth)	Discharge	Presence of SARS-CoV-2 nucleic acid in the breast milk of COVID-19 mother.
Schoenmakers et al. (60)	1	30	3 days after delivery	0	2	NA	RT-PCR	1	1,880	31	1	4	0	1	NICU admission	One mother with a positive oropharyngeal swab for SARS-CoV-2 RNA was negative for breast milk and all neonatal samples.
Sharma et al. (61)	41	26–29^l^	NA	0	23	Breastfeeding, *n* = 23	RT-PCR	44	2.60 ± 0.57	28–36, *n* = 6; >37, *n* = 37	NA	<7, *n* = 5; >7, *n* = 39	2	42	Well	The possibility of vertical transmission is almost negligible.
Luo et al. (62)	14	30.5 ± 5.2	1 and 15 days after delivery	0	14	Breastfeeding, *n* = 1; artificial feeding, *n* = 13	RT-PCR	14	2,450–3,800	34–41	7–8	8–9	8	6	Well	All breast milk samples were negative for the detection of SARS-CoV-2.
Dong et al. (63)	1	33	1 day after delivery	0	1	Breastfeeding	RT-PCR	1	2,950	38.3	9	10	0	1	Discharge	The breast milk sample collected from the infected mother was negative for SARS-CoV-2 by RT-PCR.
De Socio et al. (64)	1	33	48 h after delivery	0	1	Breastfeeding	RT-PCR	1	NA	40	10	10	0	1	Well	Under strict infection control measures, direct breastfeeding is recommended for asymptomatic COVID-19 mothers.
Young et al. (65)	47	29.9 ± 4.4 (20–38)	Day 0, 3, 7, 10, 28, and 90.	0	47	Breastfeeding, *n* = 36	RT-PCR	47	2,735–4,621	36–41	NA	NA	0	47	NA	No SARS-CoV-2 mRNA was detected in human milk samples.
Calabretto et al. (66)	12	27.2	At the time of delivery	0	12	NA	RT-PCR	NA	NA	NA	NA	NA	NA	NA	No death reported	SARS-CoV-2 appears not to be transmitted via breast milk.
Pace et al. (67)	64	30–36	Between day 1 and 106	0	316	Breastfeeding, *n* = 19	RT-PCR	20^m^	NA	Less than 24 months old	NA	NA	7^m^	13^m^	No death reported	Milk produced by women with COVID-19 showed no sign of SARS-CoV-2.
Bäuerl et al. (68)	60	34.8 ± 4.6	Between day 1 and 206	0	60	Breastfeeding, *n* = 35	RT-PCR	55	3,247 ± 519	38.1–40.6	NA	NA	0	55	No death reported	The breast milk of women with COVID-19 absents SARS-CoV-2 RNA.
Walczak et al. (69)	1	42	NA	0	1	NA	RT-PCR	1	3,770	39.4	9	9	0	1	Discharge	SARS-CoV-2 RNA was not detectable in the breast milk of pregnant women with COVID-19, but there were detectable antibodies.
Yu et al. (70)	1	32	On the 2nd, 9th, 16th and 19th day after mothers’ SARS-CoV-2 positive test	0	2	Breastfeeding	RT-PCR	1	10,000 (13 months postpartum)	13 months (postpartum)	NA	NA	1	0	Discharge	There was no evidence of mother-to-child transmission of the virus through breastfeeding. Breastfeeding is safe.
Gao et al. (71)	14	31	17–22 days after maternal symptoms onset	0	12	Breastfeeding, *n* = 4; ND, *n* = 10	RT-PCR	14	3,224	38	NA	NA	0	12^n^	Well	Breastfeeding has a low risk of transmitting SARS-CoV-2.
Fenizia et al. (72)	31	30 (15–45)	5 days after delivery	1	10	Breastfeeding, *n* = 29	RT-PCR	31	2,180–4,165	39	NA	<7, *n* = 1	2	29	UICU, *n* = 2; no death reported.	There is a detectable SARS-CoV-2 genome in breast milk samples.
Pace et al. (73)	18	34.2 ± 4.7	12.0 ± 8.9 days after the onset of symptoms	0	37	Breastfeeding, *n* = 5; mixed feeding, *n* = 13	RT-PCR	18	3,372 ± 560	38.6 ± 1.7	NA	NA	2^p^	4^p^	Well	The data do not support mother-to-infant transmission of SARS-CoV-2 via milk.

COVID-19, coronavirus disease 2019; SARS-CoV-2, severe acute respiratory syndrome coronavirus 2; RT-PCR, reverse transcription-polymerase chain reaction; NICU, neonatal intensive care unit; ND, not detailed.

^a^ Interquartile range.

^b^ Gestational age of pregnant women who delivered in the maternal sample.

^c^ Only eight cases were tested.

^d^ One infant died of a disease unrelated to COVID-19.

^e^ The six breast milk samples in this study were all from the same mother.

^f^ The sample of the positive breast milk was retested the next day.

^g^ The three breast milk samples were all from the same mother.

^h^ The 15th day after birth.

^i^ The first 3 days after birth.

^j^ Two newborns positive on the second day after birth.

^k^ Immediately after birth.

^l^ The age of the 2 pregnant women who delivered.

^m^ Only 20 infants were tested.

^n^ Infants RT-PCR results: negative, *n* = 12; NA, *n* = 2.

^o^ The number presented here is the number of mothers, not the number of breast milk.

^p^ Only 6 infants were tested.

^q^ The four breast milk samples were all from the same mother.

Of these 66 studies, 17 reported positive results for SARS-CoV-2 nucleic acid in breast milk samples (4, 6, 11, 14, 17, 18, 29, 32, 33, 36, 38, 39, 46, 47, 55, 59, 72), but only two studies monitored viral activity (6, 47). The remaining 49 studies showed negative results for SARS-CoV-2 in breast milk. Thirty-eight studies reported full or partial use of breastfeeding for newborns. Of the 17 studies that were positive for SARS-CoV-2 RNA in breast milk, 179 newborns in 11 studies were given breast milk, and only 18 (10%) had a positive nasopharyngeal swab result (one study did not report a positive result in newborns). Notably, 53 (15.2%) of the 16 studies with negative breast milk samples had positive nasopharyngeal swabs in newborns. Interestingly, three newborns in three studies of 22 formula-fed newborns also showed positive nasopharyngeal swab results (11, 31, 46). No deaths were reported in all 1,346 neonates.

### 3.2. Results of SARS-CoV-2 antibody detection in breast milk

Twenty-nine studies reported results of antibody testing against SARS-CoV-2 in at least 1,279 breast milk samples from mothers with a confirmed diagnosis of COVID-19 (9, 10, 62–88), and the majority of mothers included in these studies had mild disease severity. All 29 studies showed the presence of at least one of the three antibodies, IgA, IgG and IgM, in breast milk. Twenty-seven of these studies reported the method of detection, and 23 studies used an ELISA assay. The longest duration of antibodies was ten months (80). Twenty-three studies found IgA antibodies in breast milk samples, 17 studies found IgG antibodies in breast milk samples, and 11 studies reported the presence of IgM antibodies in breast milk. Six studies indicated that all three antibodies, IgA, IgG and IgM, were detected in breast milk (10, 68, 69, 79, 87, 88). Nine studies also reported serum antibodies in newborns born to mothers, five studies reported the presence of at least one antibody in the serum of newborns (63, 70–72, 88), most of whom were breastfed, four studies reported negative serum antibodies in newborns (62, 69, 75, 78), with one study detecting IgA antibodies in the saliva of newborns (75). Table 3 presents a summary of studies in which antibodies against SARS-CoV-2 were detected in human milk.

**Table 3:** Characteristics of included studies of detection anti-SARS-CoV-2 antibodies in human breast milk.

Study	Breast milk samples (n)^a^	Illness severity	Detection method	Duration antibody persisted from onset of COVID-19 until end of study	Detection results			Infant serum	Related findings	Feeding method
					IgA	IgG	IgM			
Demers-Mathieu et al. (10)	7	NA	ELISA	NA	100%, 7/7	100%, 7/7	100%, 7/7	NA	Breast milk of women with COVID-19 secretes large amounts of antibodies against SARS-CoV-2.	NA
Luo et al. (62)	14	Mild	ELISA	NA	ND	Negative	28.6%, 4/14	IgG (−), IgM (−)	Presence of IgM against SARS-CoV-2 in breast milk of mothers after SARS-CoV-2 infection.	Breastfeeding, *n* = 1; Artificial feeding, *n* = 13
Dong et al. (63)	6^b^	Mild	ELISA	42 days	Positive	Positive	ND	IgG (+)	IgG and IgA in breast milk may be able to provide newborns with immune protection.	Breastfeeding
De Socio et al. (64)	1	Mild	IgG/IgM fast assay	NA	ND	Positive	Weakly positive	NA	IgG and IgM antibodies present in the breast milk of mother infected with SARS-CoV-2.	Breastfeeding
Young et al. (65)	47	NA	ELISA	70 days	100%	100%	ND	NA	The IgA and IgG responses were still present in breast milk samples 90 days after SARS-CoV-2 infection.	Breastfeeding, *n* = 36
Calabretto et al. (66)	12	NA	ELISA	NA	66%, 8/12	Negative	Negative	NA	Infected mothers can breastfeed their newborns safely.	NA
Pace et al. (67)	316	NA	ELISA	2 months or longer	75%, 237/316	ND	ND	NA	Rapid, powerful, and long-lasting anti RBD specific IgA responses are produced in most breast milk.	Breastfeeding, *n* = 19
Bäuerl et al. (68)	60	NA	ELISA	226 days	65.2%–87.5%	47.8%–87.5%	52.90%	NA	Women with COVID-19 have IgA, IgG and IgM antibodies in their breast milk. The presence of Igs suggests that breast milk may have a protective effect on newborns.	Breastfeeding, *n* = 35
Walczak et al. (69)	1	Mild	In-house microsphere immunoassay	NA	Positive	Positive	Positive	Negative	SARS-CoV-2 IgG, IgM, and IgA antibodies were detected in the milk of COVID-19 patient.	NA
Peng et al. (9)	44	Mild, *n* = 15; asymptomatic, *n* = 9	ELISA	3–79 days	ND	Negative	47.7%, 21/44	NA	Some (21/44) breast milk samples were positive for IgM.	Breastfeeding, *n* = 14; formula fed, *N* = 10
Yu et al. (70)	4	Mild	NA	16 days	ND	100%, 2/2	Negative	IgG (+); IgM (+)	SARS-CoV-2 nucleic acid was not detected in breast milk, but anti-SARS-CoV-2 antibody was detected.	Breastfeeding
Gao et al. (71)	12	Mild	Chemiluminescence immunoassay	28 days	ND	17%, 2/12	17%, 2/12	IgG (+), *n* = 3; IgM (+), *n* = 1	Infants can passively acquire antibodies against SARS-CoV-2 by ingesting breast milk.	Breastfeeding, *n* = 4, 10 ND
Fenizia et al. (72)	11	Mild, *n* = 27; severe, *n* = 4	NA	NA	ND	Negative	10%, 1/10	IgM (+), *n* = 1	Specific anti-SARS-CoV-2 IgM and IgG antibodies were present in the breast milk samples.	Breastfeeding, *n* = 29
Pace et al. (73)	37	mild-to-moderate	ELISA	NA	76%, 26/34	80%, 22/27	ND	NA	The data support the recommendations to continue breastfeeding during mild-to-moderate maternal COVID-19 illness.	Breastfeeding = 5, mixed feeding = 13
Juncker et al. (74)	82	Mild	ELISA	70 days	67%, 12/18	ND	ND	NA	The IgA antibody is present in the breast milk of COVID-19 mothers.	Breastfeeding, *n* = 7
Conti et al. (75)	48 h, *n* = 6; 2 months, *n* = 10	Mild	ELISA	60 days	Positive	ND	ND	IgA (-), IgG (-); IgA (+) in Saliva	IgA in breast milk may passively protect newborns.	Breastfeeding, *n* = 17
van Keulen et al. (76)	29	Mild	ELISA	13 weeks	83%, 24/29	ND	ND	NA	IgA present for at least 13 weeks from symptom onset.	NA
Lebrão et al. (77)	2	Severe	ELISA	6 days	100% 2/2	ND	ND	NA	SARS-CoV-2IgA in the breast milk of women infected with COVID-19 may be associated with passive immunity in infants.	Breastfeeding
Favara et al. (78)	2	Mild	Rapid lateral flow point-of-care IgG test and Luminex test	195 days	Positive	Positive	ND	IgG (-), IgA (-)	Strongly neutralizing IgA and IgG antibodies were present in breast milk after SARS-CoV-2 infection and were able to persist for 6.5 months.	Breastfeeding
Fox et al. (79)	8	NA	ELISA	NA	80%	67%	67%	NA	There is strong sIgA-dominant SARS-CoV-2 immune response in human milk after infection in the majority of individuals.	NA
Juncker et al. (80)	165	Mild	ELISA	10 months	59%, 98/165	ND	ND	NA	The high prevalence of antibodies in human milk might lead to passive immunity in breastfed infants and may serve as protection against COVID-19.	NA
Szczygioł et al. (81)	72	NA	ELISA	229 days	86.1%, 62/72	84.7%, 61/72	ND	NA	SARS-CoV-2 IgA and IgG antibodies present in the breast milk of COVID-19 recovered women.	Breastfeeding, *n* = 41
Bode et al. (82)	21	NA	ELISA	8 months	42.9% (9/21), *N* fragment; 23.9% (5/21), S1/S2 fragment	42.9% (9/21) responded to *N* fragment, 23.9% (5/21) responded to S1/S2 fragment.	ND	NA	Individual COVID-19 cases had diverse and unique milk IgA profiles following the onset of symptoms.	NA
Bobik et al. (83)	41	Mild, *n* = 30	ELISA	3 months	100% (41/41) responded to sIgA1; 33% (14/41) responded to sIgA2	Negative	ND	NA	Human milk from COVID-19 convalescents possesses a protective effect against SARS-CoV-2 infection.	NA
Duncombe et al. (84)	2	Mild	ELISA	6 months	100%, 2/2	Negative	Negative	NA	Breastfeeding mothers produced a durable IgA response for up to six months following COVID-19 infection.	Breastfeeding
Juncker et al. (85)	66	NA	ELISA	5 months	From 87% to 100%^c^	Systemic positive	ND	NA	Human milk from SARS-CoV-2 convalescent lactating mothers contains specific IgA antibodies for at least 5 months post-infection.	Breastfeeding, *n* = 19
Dutra et al. (86)	165	Mild, *n* = 62; moderate, *n* = 24; severe, *n* = 10	ELISA	69 days	70.9%, 117/165	1.2%, 2/165	ND	NA	The presence of anti-SARS-CoV-2 IgA in colostrum was detected in more than two-thirds of the women evaluated and was associated with a lower frequency of clinical symptoms in their newborns.	Breastfeeding, *n* = 103
Narayanaswamy et al. (87)	15	Mild	ELISA	NA	73%, 11/15	73%, 11/15	33%, 5/15	NA	SARS-CoV-2 specific antibodies have been detected in the breast milk of women with COVID-19.	NA
Wachman et al. (88)	31	NA	ELISA	At least 6 weeks	Positive	Positive	Positive	IgA (+), IgG (+), IgM (+)	Antibodies produced by pregnant women infected with COVID-19 will transfer to the baby. There is a strong correlation between breast milk and infant IgG levels.	Breastfeeding, *n* = 29

COVID-19, coronavirus disease 2019; SARS-CoV-2, severe acute respiratory syndrome coronavirus 2; ELISA, enzyme linked immunosorbent assay; RT-PCR, reverse transcription-polymerase chain reaction; ND, not detected; NA, not available; IgA, immunoglobulin A; IgG, immunoglobulin G; IgM, immunoglobulin M.

^a^ All breast milk samples were obtained from COVID-19 mothers who were confirmed by RT-PCR.

^b^ One breast milk was tested for nucleic acid and six breast milk was tested for antibodies.

^c^ At months 1, 2, 3, 4 and 5 after symptom onset, 100% (6/6), 94% (17/18), 90% (18/20), 87% (13/15) and 100% (6/6) of samples contained SARS-CoV-2 IgA antibodies, respectively.

## 4. Discussion

### 4.1. Is SARS-CoV-2 present in breast milk?

Of the 66 studies examining the presence of SARS-CoV-2 in breast milk samples, 17 studies reported positive results for SARS-CoV-2 nucleic acid in 38 breast milk samples. Zhu et al. (17) and Thanigainathan et al. (39) retested breast milk collected from the same mother the next day of the positive samples and showed different results. The results of Zhu et al. suggested that the breast milk was still positive for SARS-CoV-2, while the results of Thanigainathan et al. were negative. Similarly, Tam et al. (32) repeated the test on breast milk samples from the same mother on day 5 and day 15 after the onset of symptoms, and the results were positive. In contrast, Hinojosa-Velasco et al. (11) reported a mother whose breast milk showed a positive result on day 1 after delivery but a negative result when her breast milk sample was retested on day 13 after delivery. A report from Groß et al. (4) of 11 breast milk samples from two mothers found positive breast milk samples from the same mother on four consecutive days. Costa et al. (36) also reported three positive results for SARS-CoV-2 in six breast milk samples from one woman. Kilic et al. (38) investigated breast milk samples from 15 COVID-19 mothers, and SARS-CoV-2 RNA was detected in the breast milk of four mothers. A study from Italy tested ten breast milk samples collected from the same mother during the first five days after the birth of her newborn, and three samples were positive for SARS-CoV-2 RT-PCR (46). The remaining 49 studies, including at least 1,229 breast milk samples, did not detect SARS-CoV-2 RNA. These data suggest that SARS-CoV-2 RNA is indeed present in the breast milk of COVID-19 mothers.

### 4.2. Is breast milk a carrier of vertical transmission from mother to child?

#### 4.2.1. The presence of SARS-CoV-2 nucleic acid in breast milk does not represent a replication-competent virus

Although SARS-CoV-2 RNA was detected in 38 breast milk from 17 studies, only two studies monitored viral activity (6, 47). Krogstad et al. (47) performed viral cultures on 160 breast milk samples and did not detect the virus in any culture, although SARS-CoV-2 RNA was detectable in 9.2% of these milk samples. Notably, they artificially added virus to breast milk samples from control experiments and infectious SARS-CoV-2 could be cultured despite several freeze-thaw cycles. Chambers et al. (6) added replication-competent SARS-CoV-2 to two breast milk samples that were pasteurized. The authors failed to detect SARS-CoV-2 RNA or culturable virus in the Holder pasteurized breast milk samples. In contrast, the two unpasteurized breast milk samples from the control group were found to be positive for viral RNA. These studies suggest that SARS-CoV-2 virus particles detected in breast milk are not infectious. In fact, there are no reports of transmission of SARS-CoV and Middle East Respiratory Syndrome coronavirus (MERS-CoV) through breast milk.

#### 4.2.2. Most children breastfed from nucleic acid-positive breast milk have negative results

Of the 179 children given breast milk in the 17 breast milk SARS-CoV-2 positive studies, only 18 (10%) of these children had positive nasopharyngeal swab SARS-CoV-2 nucleic acid results and the majority (90%) of newborns had negative nasopharyngeal swab results.

#### 4.2.3. Children breastfed from nucleic acid-negative breast milk show positive results for SARS-CoV-2

Of the 26 studies in which breast milk samples tested negative for nucleic acid, 219 children were given breast milk, and 11 of these studies found 24 children with positive nasopharyngeal swab nucleic acid test results for SARS-CoV-2.

#### 4.2.4. Feeding method is not breastfeeding despite the child being SARS-CoV-2 nucleic acid positive

Results from three of the eight formula-fed studies showed that three children tested positive for SARS-CoV-2 on nucleic acid from nasopharyngeal swabs (11, 31, 46).

#### 4.2.5. Positive mother and positive child, but breast milk negative

The mothers included in this review were all SARS-CoV-2 positive mothers, and 50 children born to these positive mothers in 16 studies turned out to be SARS-CoV-2 positive, but the breast milk of these mothers tested negative for SARS-CoV-2.

In addition, the largest cohort study published to date showed no evidence of vertical mother-to-child transmission when newborns were roomed with their mothers and breastfed. On the other hand, mother-child separation does not guarantee a virus-free environment for the infant.

These findings provide strong evidence that there is no necessary link between breastfeeding and a child being nucleic acid positive, suggesting that breast milk is not a source of vertical transmission. All this evidence supports the view that mothers infected with SARS-CoV-2 do not put their newborns at additional risk of infection through breastfeeding.

### 4.3. What are the reasons for positive results?

#### 4.3.1. Contaminated or exposed to a third party infected with SARS-CoV-2

1. The first is respiratory contamination from the mother

Mothers do not wear masks when collecting breast milk samples or breastfeeding, which increases the likelihood that breast milk or the newborn will be contaminated with the mother's respiratory secretions, especially during the acute symptomatic phase when the mother's respiratory viral load is extremely high (89).

2. The second is pollution from the environment

Because breastfeeding involves a range of intimate behaviors between mother and infant, including hand contact and skin-to-skin contact with the breast and contact with the surface of the container holding the milk, the risk of environmental contamination of breast milk or the newborn is possible (90).

3. Exposure to a SARS-CoV-2 infected third party

Newborns were infected with SARS-CoV-2 after delivery through close contact with infected family members (43), especially considering that in these cases, parents, relatives, caregivers of newborns, and individuals in their communities were diagnosed with COVID-19 and were susceptible to horizontal human-to-human transmission (91).

We believe that even if the detectable SARS-CoV-2 RNA in breast milk samples is the result of contamination, this is still an important finding because it is a potential and unanticipated source of exposure for uninfected infants. This emphasizes the importance of proper breast hygiene before feeding.

#### 4.3.2. Virus shedding

SARS-CoV-2 enters host cells by binding to the ACE2 receptor (92, 93). The ACE2 receptor is expressed in both female reproductive organs and breast tissue (94). Although ACE2 receptor expression is extremely low in breast tissue (only 5%), SARS-CoV-2 could theoretically be present in any tissue with an ACE2 receptor and, therefore, SARS-CoV-2 may still be shed from the breast milk of infected mothers during lactation.

#### 4.3.3. Reflux infection

Human milk has the potential to be contaminated with SARS-CoV-2 RNA from the infant's oropharynx to the breast. If the infant is infected earlier than the infant's parents, there is a possibility of “reverse” vertical transmission from the infant to the mother, a phenomenon that has been observed in other pathogens such as HIV (95, 96) and Ebola (97). In this case, one possible mechanism of maternal infection is retrograde flow, in which milk and saliva move from the infant's mouth back to the mammary gland during suckling.

#### 4.3.4. Associated with higher viral load

It is known that some viruses can be transmitted through breast milk and that higher serum viral load can increase the risk of transmission (98). In the case of HIV and HTLV-1, the level of the virus in breast milk correlates with the amount of virus in the whole body (2, 99, 100).

In addition to this, Bastug et al. (59) believe that the method of detection of the virus in breast milk, the timing of sample collection, and the transportation and storage of the samples are all potential factors that could lead to a positive result.

### 4.4. Relationship between positive results and neonatal outcome

Although 17 studies reported the presence of detectable SARS-CoV-2 RNA in breast milk samples, however, it is reassuring, that longitudinal follow-up of most studies showed no adverse outcomes in infants who still continued to be breastfed, including those in whom viral RNA was detectable on the skin of the mother's breast (73). Of the 1,346 newborns included, only three were admitted to the NICU (60, 72). Notably, in the context of the COVID-19 pandemic at the time, most of the newborns in the study were admitted to the NICU for isolation and observation rather than for any absolute indication. No deaths due to COVID-19 were reported in all neonates.

### 4.5. Negative results and interpretation

There are several possible reasons why the majority of studies (74%) failed to detect SARS-CoV-2 RNA in the breast milk of COVID-19 mothers. One of the most important reasons could be due to the extremely low level of ACE2 expression in the breast (94, 101, 102). Indeed, ACE2 needs to be co-expressed with protease, TMPRSS2, or CTSB/L to activate the S protein and promote the entry of SARS-CoV-2 into host cells. However, only 5% of mammary cells express ACE2 (103). The second reason is the antiviral mechanism of SARS-CoV-2 by specific substances in breast milk. Breast milk contains whey protein (104), lactoferrin (105), and mucin (106), which can block virus entry and replication by binding to various receptors of SARS-CoV-2. In addition, the presence of antibodies in breast milk, especially IgA antibodies, which have neutralizing activity against SARS-CoV-2, is also believed to be one of the reasons for the absence of SARS-CoV-2 in breast milk (67). Third, if breast milk samples are collected farther back in time from the time of infection, the viral load in breast milk will be lower, especially for asymptomatic mothers, who may not be able to detect SARS-CoV-2 RNA in breast milk because it is difficult for them to determine the onset of infection.

### 4.6. Is anti-SARS-CoV-2 antibody present in breast milk?

#### 4.6.1. The presence of antibodies in breast milk and the factors influencing them

All 29 studies that reported antibody test results indicated the presence of at least one of three antibodies in breast milk, IgA, IgG and IgM, primarily IgA but also IgG and IgM against SARS-CoV-2 RBD. These studies showed the presence of different types of antibodies and different rates of positivity in breast milk, while four studies indicated the presence of one or more antibodies in all (100%) of their respective breast milk samples (10, 55, 70, 77). In a multicenter study from the United States that examined 316 breast milk samples for levels of anti-RBD IgA, Pace et al. (67) found that 75% (*n* = 316) of the milk samples contained anti-RBD IgA for at least two months. This is by far the largest study of breast milk sample size. A large prospective cohort study by Juncker et al. (80) showed that of 165 participants who had confirmed SARS-CoV-2 infection by PCR, 98 (59%) had SARS-CoV-2 specific IgA antibodies in their breast milk. Such antibodies were still present at least ten months after infection. In Brazil, Dutra et al. (86) conducted a cross-sectional study of 165 participants infected with SARS-CoV-2 during pregnancy and their newborns, collecting postpartum colostrum samples from mothers. The results showed that 117 (70.9%) women were positive for anti-SARS-CoV-2 IgA in colostrum and confirmed that the presence of anti-SARS-CoV-2 IgA in colostrum was independently associated with lower clinical signs in newborns. Two (1.2%) participants were also found to be positive for anti-SARS-CoV-2 IgG in colostrum. These two studies are the largest samples of mothers included to date (80, 86). The study by Bode et al. (82) showed that detectable IgA antibodies were found in breast milk even eight months after onset. IgM antibodies were also present in breast milk, albeit at lower concentrations (9, 10, 62, 68, 87). In general, IgM and IgA-like responses often occur earlier after the onset of the disease, while IgG responses occur later (107). It is known that antibody abundance in breast milk is variable and there is a high degree of intra- and inter-individual variability in the three antibody classes (108). A study by Fox et al. (79) has demonstrated much higher titers of IgA than expected in 12/15 milk samples. The difference between IgA and IgG may be due to the fact that systemic IgG-secreting B cells are shorter-lived than IgA-secreting B cells in the submucosa of the breast. It is likely that antibody secretion also depends on the time elapsed after the onset of the disease, and that antibody levels in breast milk decline over time (84, 109). Those participants who showed only IgA antibodies in breast milk were more likely to be enrolled immediately after SARS-CoV-2 infection, whereas those who showed only IgG antibodies in breast milk were more likely to be enrolled some time after infection. Breast milk samples were collected closest to the time point of SARS-CoV-2 infection where antibody levels were likely to be highest. In addition, the severity of symptoms may have influenced the residence time of antibodies in breast milk, with more severe SARS-CoV-2 infections resulting in immune responses with higher antibody levels (110, 111).

#### 4.6.2. Transfer of antibodies from breast milk

Large amounts of Igs in the mother's body can be transmitted to the newborn through breast milk (112, 113). IgG antibodies are a possible indication of immunity or resistance. Of the 29 included studies that reported breast milk antibody test results, five showed that neonates were seropositive for antibodies to SARS-CoV-2 (63, 70–72, 88). Interestingly, Conti et al. (75) also detected the presence of IgA antibodies in the saliva of newborns. In China, Gao et al. (71) conducted an observational clinical analysis of 14 mother-infant pairs and found that three of the breast milk samples tested positive for SARS-CoV-2 IgM or IgG, and the corresponding three newborns fed by breast milk tested negative for SARS-CoV-2 RNA at birth, while they tested positive for SARS-CoV-2 IgG, and one of the neonates also tested positive for IgM. No SARS-CoV-2 nucleic acid was detected in breast milk samples at different stages. This suggests that specific antibodies to SARS-CoV-2 can be transferred to the infant through breast milk and provide potential protection to the neonate. Similarly, the studies of Dong et al. (63) and Fenizia et al. (72) support this idea. At present, it is unclear which factors affect the efficiency of the transfer of these maternal antibodies, and further studies are needed.

#### 4.6.3. Protective effect of breast milk antibodies

It is well known that antibodies can bind to the RBD of the surface spike protein of SARS-CoV-2, preventing the virus from binding to the ACE2 receptor of the target cell (114). This implies that in the context of COVID-19, antibodies are able to limit virus transmission. The secretory immunoglobulin A (sIgA) of anti-SARS-CoV-2 neutralizes SARS-CoV-2 before they reach and bind to epithelial cells. Secretory IgA acts directly on the mucosal surface to inhibit microbial binding to host epithelial cell receptors and to trap pathogenic microorganisms in mucus, enhancing ciliary activity and thereby eliminating invading pathogens (115, 116), providing durable passive immunity to newborns and infants (117). In addition to IgA, IgG in breast milk can attack viral envelope glycoproteins and provide the body with potent systemic antibodies. IgG also acts as an antibody that degrades intracellular viruses by binding to a crystallizable cytosolic fragment motif containing −21 tripartite receptor (TRIM21) (118). These antibodies can be passed on to offspring through breastfeeding, preventing or reducing the severity of disease in newborns. It has been found that even partially degraded IgA, IgG and IgM antibodies, if their antigen-binding fragment (Fab) fraction remains intact, still function and bind to the antigen, leading to target degradation and elimination from the intestine (119). This suggests that any neutralizing SARS-CoV-2 targeting antibodies present in breast milk may have a protective effect on infants receiving breast milk. In addition, intestinal Fc receptors may transport antibodies from the gut to the vascular circulation, further aiding systemic defense. IgA spiking antigen immune complexes would also stimulate active immunity and contribute to the maturation of the infant's immune system (75). This will facilitate the protection of the newborn from a variety of viral pathogens. This active form of protection of human milk against viruses cannot be provided to the infant through artificial formula.

Typically, maternal IgG antibodies are retained in the infant for more than six months, and infants who are unable to produce immunoglobulins are protected by maternal antibodies for up to 12 months after birth (120). Intriguingly, some studies suggest that antibodies transferred to newborns through breast milk are lower than expected (121) and decline in the infant faster (122) and for a shorter duration than expected (63).

These data highlight that preferential transfer of SARS-CoV-2 specific IgA and IgM and/or IgG in breast milk to newborns can create a non-pathological, but highly protective barrier against COVID-19 disease in breastfed infants. Unfortunately, some studies have focused on the presence and characteristics of breast milk antibodies, and incomplete information about newborns has weakened the support for the protective effect of antibodies on infant and child health. Also, these studies did not consider the effect of drugs used to treat COVID-19 on breast milk antibodies. Larger sample sizes and long-term follow-up studies with long-term follow-up of antibody class, duration, and titer changes are needed to better understand the association of SARS-CoV-2 antibodies in milk with COVID-19 outcomes in infants.

## 5. What are the benefits of breastfeeding?

Breast milk is rich in nutrients (123) and since it requires no preparation and is always at the right temperature, it helps to provide a complete, balanced, adequate, and proper diet (124). Not only is it the gold standard source of nutrition for infants, but it is the primary source of passive and active immunization for newborns (81), and breastfeeding can reduce neonatal mortality (125). The role of breastfeeding in protecting infants from infection is also crucial. The immaturity of an infant's immune system at birth increases the risk of infection from external factors, including viruses and bacteria (126), and breastfeeding can significantly reduce the risk of infection in infants (127). Breast milk contains antibodies, linoleic acid (128, 129), milk fat globule membranes, human milk oligosaccharides, osteopontin, and other antiviral components (130, 131). These nutrients bind to some of the receptors required for viral entry into host cells, limiting the ability of viruses to enter and achieving antiviral [including papillomavirus (132), human immunodeficiency virus (133), rotavirus (134), chikungunya virus (135) and Zika virus (135)], antiparasitic (136) and antifungal effects (137).

In addition, the large presence of bioactive mediators in breast milk [IL-1b, IL-2, IL-6, IL-8, IL-10, IL-12, IL-18, IFN-γ, TNFα, TGF-b, granulocyte colony-stimulating factor (G-CSF), macrophage colony-stimulating factor (M-CSF), granulocyte-macrophage colony-stimulating factor (GM-CSF)] (138, 139), which can compensate for the lack of cytokine pool in newborns. On the other hand, the benefits of breast milk are much broader, considering the emotional, cognitive, psychological, socioeconomic, and environmental development of the child and mother (140, 141). Mother-infant separation hurts the physical and mental health of both mother and infant (142), and separation of mother and infant after birth is considered a source of stress, leading to an increased incidence of developmental problems in children (143). For mothers, early cessation of breastfeeding is a significant cause of maternal depressive symptoms in the postpartum period (144). In addition, a reduced risk of breast cancer or reproductive system cancers has been observed in breastfeeding women (145). Breastfeeding mothers are less likely to develop hypertension, diabetes, hyperlipidemia, cardiovascular disease, and metabolic syndrome (146, 147).

## 6. Strengths and limitations

To our knowledge, this is the largest systematic review of SARS-CoV-2 and its antibodies in breast milk to date, with the largest number of included studies, the most comprehensive assessment parameters, and the most thorough assessment of virological evidence. The studies that examined nucleic acids included different time points of breast milk collection, included cases at different gestational weeks, and had more representative findings. The geographic distribution of the included study population was globally representative (21 countries including Arab countries). In studies examining antibodies, the presence and effectiveness of antibodies in breast milk against the virus were linked to the clinical outcomes of newborns. Our review answers a series of real-world questions of great relevance, particularly those that physicians and mothers are eager to answer. These data will have implications beyond the epidemic, providing objective, evidence-based data for developing optimal strategies for breastfeeding and maternal and infant management in similar epidemic pandemics in the future.

Our study also has some limitations. First, most studies did only qualitative SARS-CoV-2 RNA analysis and lacked precise viral load assessment, and only two studies performed cell culture. Second, most studies were retrospective, observational, and cross-sectional, and some studies did not focus on the presence of nucleic acids or antibodies but simply reported nucleic acid or antibody results, and these limitations limited our ability to study the long-term health effects of breastfeeding on infants born to SARS-CoV-2-infected mothers. Third, the small size of the sample for most of the studies, which included 33 case reports and nine case series. Despite repeatedly reading and analyzing the included literature, we could not confirm that we had excluded all potentially repeatedly reported cases. Also, there is a risk of publication bias given that COVID-19-positive cases in infants and breast milk are most likely to be reported and published. Finally, given the importance of summarizing all available cases, we did not assess the quality of the studies included in this review.

## 7. Conclusion

In conclusion, regardless of whether the breast milk of confirmed COVID-19 mothers was positive or negative for SARS-CoV-2 RNA, direct breastfeeding did not pose an additional risk of infection to the neonate, and in any case, neonatal outcomes were favorable, with no neonatal COVID-19-related deaths and good health outcomes for all infants in all studies included in this review. Also, breast milk secreted by infected mothers is a beneficial source of anti-SARS-CoV-2 antibodies that neutralize the activity of SARS-CoV-2. Not only does direct breastfeeding not exacerbate the severity of the disease, but it may provide passive immune protection to the infant. In addition, direct breastfeeding would benefit the mother by reducing depression and anxiety in the mother. Notably, given the potential for respiratory or contact transmission (horizontal transmission) of SARS-CoV-2 during breastfeeding, breastfeeding should be conducted under appropriate infection control guidelines.

## Data Availability

The original contributions presented in the study are included in the article/**Supplementary Material**, further inquiries can be directed to the corresponding author.
