# Supplementary material for: Breastfeeding vs. breast milk transmission during COVID-19 pandemic, which is more important?
Source: Front Pediatr. 2023 Sep 6;11:1253333. doi: 10.3389/fped.2023.1253333 (PMC10511770; doi:10.3389/fped.2023.1253333)
Supplement: Supplementary file 1 [file Datasheet1.docx]

**Search Strategy**

**PubMed**

#1 (COVID-19[Supplementary Concept] OR COVID-19 diagnostic testing[Supplementary Concept] OR severe acute respiratory syndrome coronavirus 2[Supplementary Concept] OR severe acute respiratory syndrome coronavirus 2[tiab] OR COVID-19[tiab] OR COVID19[tiab] OR COVID2019[tiab] OR 2019 ncov[tiab] OR 2019-nCoV[tiab] OR 2019nCoV[tiab] OR SARS COV 2[tiab] OR SARS-CoV-2[tiab] OR SARS2[tiab] OR hcov19[tiab] OR hcov2019[tiab] OR hcov-19[tiab] OR hcov-2019[tiab] OR wuhan coronavirus*[tiab] OR coronavirus disease 2019[tiab] OR coronavirus disease-19[tiab] OR SARS2[tiab] OR novel coronavirus*[tiab] OR novel corona virus*[tiab] OR new coronavirus*[tiab] OR new corona virus*[tiab] OR COVID 2019[tiab])

#2 (Breast Feeding[Mesh] OR Milk, Human[Mesh] OR Lactation[Mesh] OR colostrum[mesh] OR Breastfe*[tiab] OR breast fe*[tiab] OR breastmilk[tiab] OR breast milk[tiab] OR human milk[tiab] OR maternal milk[tiab] OR prelacteal feed*[tiab] OR lactati*[tiab] OR colostrum[tiab] OR mixed feeding[tiab] OR mother’s milk[tiab] OR expressed milk[tiab] OR milk bank*[tiab])

#3 (Vertical transmission[tiab] OR postnatal transmission[tiab] OR post natal transmission[tiab] OR maternal-infant transmission[tiab] OR adult-to-child[tiab] OR maternal-to-child[tiab] OR mother-to-child[tiab] OR MTCT[tiab] OR PMTCT[tiab])

#4 (Antibodies[tiab] OR antibodies, viral [tiab] OR immunoglobulins[tiab] OR Immunoglobulin G tiab] OR Immunoglobulin M[tiab] OR antibod* [tiab] OR immunoglobulin[tiab])

#5 (NOT (vaccine OR Vaccination))

#6 #1 AND #2 AND #3 AND #5

#7 #1 AND #2 AND #4 AND #5

#8 #5 AND #6

**MEDLINE**

#1 MeSH descriptor: [Breast Feeding[Mesh] OR Milk, Human[Mesh] OR Lactation[Mesh] OR colostrum[mesh]

#2 Breastfe*[tiab] OR breast fe*[tiab] OR breastmilk[tiab] OR breast milk[tiab] OR human milk[tiab] OR maternal milk[tiab] OR prelacteal feed*[tiab] OR lactati*[tiab] OR colostrum[tiab] OR mixed feeding[tiab] OR mother’s milk[tiab] OR expressed milk[tiab] OR milk bank*[tiab])

#3 (Vertical transmission[tiab] OR postnatal transmission[tiab] OR post natal transmission[tiab] OR maternal-infant transmission[tiab] OR adult-to-child[tiab] OR maternal-to-child[tiab] OR mother-to-child[tiab] OR MTCT[tiab] OR PMTCT[tiab])

#4 (COVID-19):ti,ab,kw OR (COVID 19):ti,ab,kw OR (COVID19):ti,ab,kw OR (SARS-CoV-2):ti,ab,kw OR (SARS CoV 2):ti,ab,kw OR (SARS2):ti,ab,kw OR (Coronavirus Disease 2019):ti,ab,kw OR (Coronavirus Disease-19):ti,ab,kw OR (Coronavirus Disease 19):ti,ab,kw OR (2019 nCoV):ti,ab,kw OR (2019 Novel Coronavirus):ti,ab,kw OR (SARS Coronavirus 2):ti,ab,kw

#5 (Antibodies[tiab] OR antibodies, viral [tiab] OR immunoglobulins[tiab] OR Immunoglobulin G tiab] OR Immunoglobulin M[tiab] OR antibod* [tiab] OR immunoglobulin[tiab])

#6 (NOT (vaccine OR Vaccination))

#7 #1 AND #2 AND #3 AND #4 AND #6

#8 #1 AND #2 AND #4 AND #5 AND #6

**EMBASE**

1# Exp breast feeding/ OR exp breast milk/ OR lactation/ OR exp colostrum/

2# ("Breastfe*" OR "breast fe*" OR "breastmilk" OR "breast milk" OR "human milk" OR "maternal milk" OR "mother’s milk" OR "prelacteal feed*" OR "lactati*" OR "colostrum" OR "mixed feeding" OR "mother’s milk" OR "expressed milk" OR "milk bank*").mp

3# ("vertical transmission" OR "postnatal transmission" OR "post natal transmission" OR "maternal-infant transmission" OR "adult-to-child" OR "maternal-to-child" OR "mother-to-child" OR "MTCT" OR "PMTCT").mp

4# ("severe acute respiratory syndrome coronavirus 2" OR "COVID-19" OR "COVID19" OR "COVID2019" OR "2019 ncov" OR "2019-nCoV" OR "2019nCoV" OR "SARS COV 2" OR "SARS-CoV-2" OR "SARS2" OR "hcov19" OR "hcov2019" OR "hcov-19" OR "hcov-2019" OR "wuhan coronavirus*" OR "coronavirus disease 2019" OR "coronavirus disease-19" OR "SARS2" OR "novel coronavirus*" OR "novel corona virus*" OR "new coronavirus*" OR "new corona virus*" OR "COVID 2019").mp

5# (Antibodies[tiab] OR antibodies, viral [tiab] OR immunoglobulins[tiab] OR Immunoglobulin G tiab] OR Immunoglobulin M[tiab] OR antibod* [tiab] OR immunoglobulin[tiab])

#6 (NOT (vaccine OR Vaccination))

#7 (#1 OR #2) AND #3 AND #4 AND #6

#8 (#1 OR #2) AND #4 AND #5 AND #6

**Cochrane Library**

#1 MeSH descriptor: [Breast Feeding] explode all trees

#2 MeSH descriptor: [Milk, Human] explode all trees

#3 MeSH descriptor: [Lactation] explode all trees

#4 MeSH descriptor: [Colostrum] explode all trees

#5 ("Breastfe*" OR "breast fe*" OR "breastmilk" OR "breast milk" OR "human milk" OR "maternal milk" OR "prelacteal feed*" OR "lactati*" OR "colostrum" OR "mixed feeding" OR "mother’s milk" OR "expressed milk" OR "milk bank*"):ti,ab,kw

#6 #1 OR #2 OR #3 OR #4 OR #5

#7 MeSH descriptor: [Infectious Disease Transmission, Vertical] explode all trees

("vertical transmission" OR "postnatal transmission" OR "post natal transmission" OR "maternal-infant transmission" OR "adult-to-child" OR "maternal-to-child" OR "mother-to-child" OR "MTCT" OR "PMTCT"):ti,ab,kw

#8 ("severe acute respiratory syndrome coronavirus 2" OR "COVID-19" OR "COVID19" OR "COVID2019" OR "2019 ncov" OR "2019-nCoV" OR "2019nCoV" OR "SARS COV 2" OR "SARS-CoV-2" OR "SARS2" OR "hcov19" OR "hcov2019" OR "hcov-19" OR "hcov-2019" OR "wuhan coronavirus*" OR "coronavirus disease 2019" OR "coronavirus disease-19" OR "SARS2" OR "novel coronavirus*" OR "novel corona virus*" OR "new coronavirus*" OR "new corona virus*" OR "COVID 2019"):ti,ab,kw

#9 "(Antibodies[tiab] OR antibodies, viral [tiab] OR immunoglobulins[tiab] OR Immunoglobulin G tiab] OR Immunoglobulin M[tiab] OR antibod* [tiab] OR immunoglobulin[tiab])

#10 (NOT (vaccine OR Vaccination))

#11 #6 AND #7 AND #8 AND #10

#12 #6 AND #7 AND #8 AND #10
